# SRF promotes gastric cancer metastasis through stromal fibroblasts in an SDF1-CXCR4-dependent manner

**DOI:** 10.18632/oncotarget.10024

**Published:** 2016-06-14

**Authors:** Juanli Qiao, Zhaojun Liu, Chen Yang, Liankun Gu, Dajun Deng

**Affiliations:** ^1^ Key Laboratory of Carcinogenesis and Translational Research (Ministry of Education/Beijing), Division of Etiology, Peking University Cancer Hospital and Institute, Beijing, 100142, China

**Keywords:** SRF, SDF1, myofibroblast, gastric carcinoma, metastasis

## Abstract

It has been suggested that the overexpression of serum response factor (*SRF*) in cancer cells may promote cancer metastasis. However, the exact pathway by which *SRF* promotes metastasis has not been clarified. Here we showed that SRF promotes gastric cancer (GC) metastasis through stromal fibroblasts in an SDF1-CXCR4-dependent manner. *SRF* expression was significantly increased in metastatic GCs compared with the non-metastatic GCs (*n*=50, *p*=0.013). Immuno-staining indicated that *SRF* was primarily expressed in a-smooth muscle actin (αSMA)-expressing periglandular fibroblasts in GCs. The conditioned medium (CM) from CCD18Co fibroblasts stably transfected with the *SRF* vector (CCD18Co-SRF) significantly enhanced migration of MKN45 gastric cancer cells. In contrast, the CM from CCD18Co fibroblasts, in which *SRF* was downregulated, inhibited mobility of MKN45 cells. Similar results were observed in cultured BGC823 cells even when they were treated with the NIH3T3-SRF CM. When MKN45 cells and *SRF*-upregulated or downregulated CCD18Co cells were simultaneously co-injected into the tail veins of NOD-SCID mice, a significant increase or decrease was, respectively, observed in the experimental pulmonary metastasis of cancer cells. Furthermore, *SRF* overexpression significantly upregulated *`SMA* and stromal cell derived factor1 (*SDF1*) expression in these fibroblasts, and an anti-SDF1 antibody or the SDF1 receptor CXCR4-specific inhibitor AMD3100 treatment completely reversed the *SRF-*enhanced migration of cancer cells. Quantitative RT-PCR demonstrated that the expression level of *SRF* was positively correlated with that of *SDF1* in 92 GC samples (*r*=0.63, *p*<0.001). In conclusion, *SRF* promote GC metastasis by facilitating myofibroblast-cancer cell crosstalk in an SDF1-CXCR4 dependent manner, and maybe a therapeutic target.

## INTRODUCTION

Gastric carcinoma (GC) is a leading cause of cancer-related deaths worldwide [1, 2]. Metastasis is found in more than half of advanced GC patients and causes the majority of cancer-related mortalities. Although this disease has been widely studied, the exact mechanism of GC metastasis has not been clarified [3].

Serum response factor (SRF) is a ubiquitously-expressed transcription factor that regulates the expression of over 200 genes [4]. It is a master regulator of myogenesis and other cell processes, including immediate early and tissue-specific gene expression, cytoskeletal organization, cell proliferation/differentiation/migration, epidermal stem cell fate decision, and angiogenesis. It acts in cooperation with c-Fos/JunB, MRTFs, E-/VE-Cadherin/β-Catenin, RhoA, and Actin in various types of cells and tumor stroma [4–14]. SRF is overexpressed in many cancers and may promote the epithelial-mesenchymal transition and the invasion/metastasis of cancer cells [15–17]. Although it has been reported that the upregulation of *SRF* expression in cancer cells promotes GC invasion/metastasis [18], *SRF* might also be involved in cancer metastasis through its activity in fibroblasts [19, 20].

Tumor stromal tissue has a profound influence on the development and progression of cancers [21]. Myofibroblasts are an important component of tumor stromal cells (sometimes termed “activated fibroblasts”), which play important roles in tumor progression [22–24]. It has been reported that SRF can upregulate αSMA expression and promote the differentiation of myofibroblasts during fibrosis [25]. However, whether SRF contributes to GC metastasis through myofibroblasts has not been studied.

Recently, we have observed that inactivation of *SRF* by hypermethylation of CpG islands in surgical margin (SM) tissues is significantly associated with a decreased risk of GC metastasis and a long overall survival in multiple patient cohorts [26]. In the present study, we further report that *SRF* is overexpressed in GC stromal myofibroblasts and promotes the invasion and migration of GC cells by facilitating myofibroblast-cancer cell crosstalk in an SDF1-CXCR4-dependent manner.

## RESULTS

### Characterization of SRF-expressing cells in gastric carcinoma tissues

**Figure 1:**
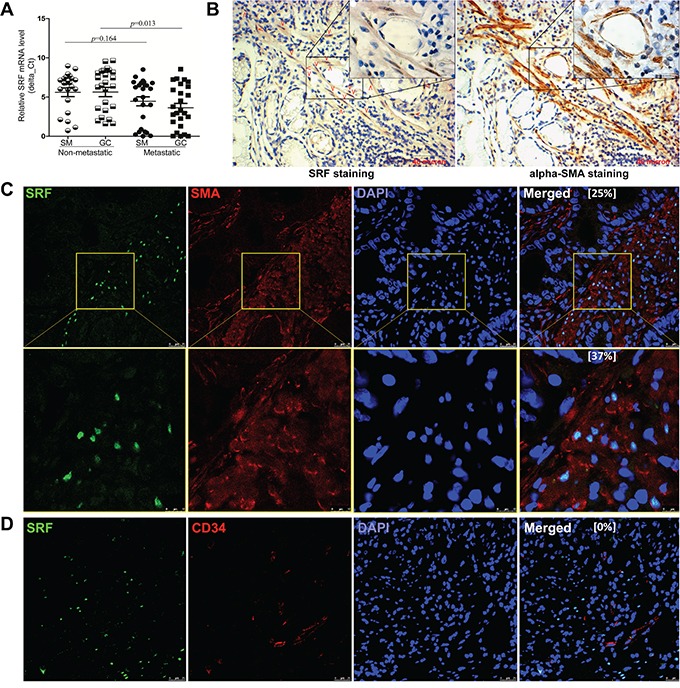
Characterization of *SRF* expression in gastric carcinoma tissues **A.** The relative *SRF* mRNA levels in 25 metastatic and 25 non-metastatic gastric carcinoma (GC) tissue samples and their corresponding surgical margin (SM) tissue samples, were determined by quantitative RT-PCR. **B.** The distribution of SRF- and αSMA-positively stained cells in serial IHC slides of GC stroma from the same gastric lamina propria region. The SRF-expressing cells were marked with red arrows. **C** and **D.** Images from indirect IHF experiments showed SRF- and αSMA-positively stained cells in a representative GC sample. Ratios of SRF-expressing αSMA- or CD34-antibody positively stained cells were showed in the merge image, respectively.

Result of qRT-PCR showed that the average *SRF* mRNA level in 25 metastatic GCs was significantly higher than that in 25 non-metastatic GCs (Figure 1A; *p*=0.013). Immnohistochemical staining (IHC) and immunohistofluorescence (IHF) were used to characterize the SRF-expressing cells in GC tissues. Unexpectedly, strong SRF signal was observed only in the nucleus of some stromal cells in both GC and SM tissues, but not in cancer cells nor in gastric mucosa epithelial cells from normal/superficial gastritis biopsies from non-cancer control subjects (Suppl. Figure 1). Nested quantitative RT-PCR analysis also showed the level of *SRF* mRNA in the laser capture micro-dissected stromal cells was two times of that in the dissected GC cells (Suppl. Figure 2). We further carried out immunostaining for both SRF and αSMA using serial slides, and found that SRF-expressing cells and αSMA-expressing cells were overlapped in the lamina propria (Figure 1B). Notably, most SRF-expressing cells co-localizes with αSMA-expressing cells and about 25% αSMA-expressing cells simultaneously express SRF (Figure 1C), but not CD34 in GC stromal tissues (Figure 1D). These results indicate that the SRF-expressing stromal cells in the mucosa lamina propria are mainly gastric periglandular myofibroblasts.

### SRF in fibroblasts promotes the migration of cancer cells *in vitro*

**Figure 2:**
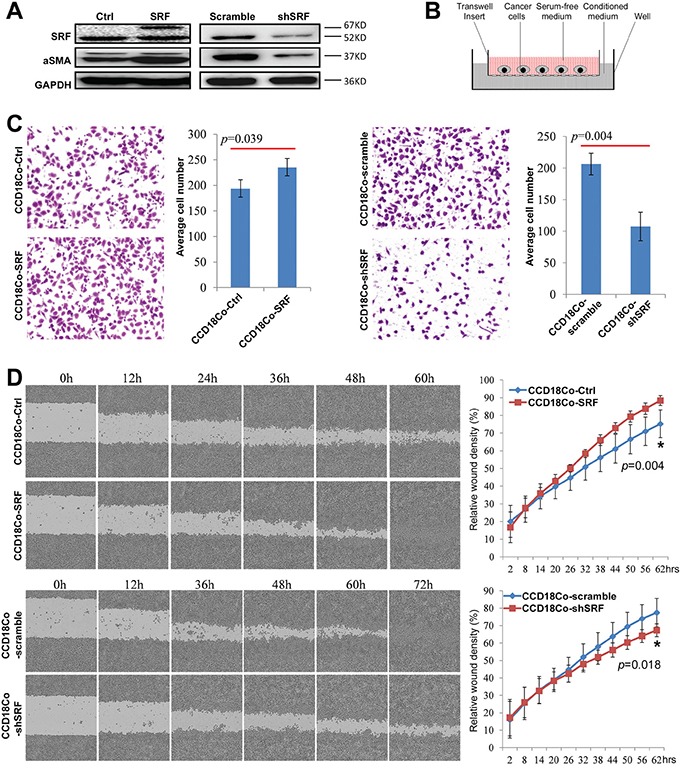
SRF in CCD18Co fibroblasts promotes the migration of cultured cancer cells *in vitro* **A.** SRF and αSMA expression levels in CCD18Co cells stably transfected with the SRF-pTRIPZ or shSRF-LV3 vector were determined by western blotting. **B.** The transwell model containing an insert used to detect the effect of various fibroblast-conditioned medium on the migration of cultured cancer cells. **C.** The migration of MKN45 cells cultured for 12 hrs with conditioned medium from CCD18Co cells with upregulation or downregulation of SRF expression were determined by the transwell assay as described in **(B)**. The individual cell numbers in 3 wells were used to calculate the average value. **D.** The migration of MKN45 cells cultured with different conditioned medium was determined using the wound-healing assay. The values from 6 wells were used to calculate the mean and SD at each time point for each treatment.

To study the effect of fibroblast *SRF* on the migration and invasion of cancer cells, we stably transfected the human fibroblast cell line CCD18Co with the human SRF-pTRIPZ vector (Figure 2A, left), collected the serum-free medium (CCD18Co-SRF) 24 hrs after doxycycline induction, and used the conditioned medium (CM) to culture MKN45 GC cells, as illustrated in Figure 2B. The migration of MKN45 cells cultured in the CM was significantly increased compared with that of MKN45 cells cultured with the control CM from CCD-8Co cells transfected with the control vector (CCD18Co-Ctrl) (Figure 2C, left). In contrast, the migration of MKN45 cells was significantly decreased when cultured in the CM from CCD18Co cells transfected with the SRF-specific shRNA vector (CCD18Co-shSRF) (Figure 2A and 2C, right). These results were further confirmed in a wound-healing assay using the IncuCyte platform (Figure 2D). Moreover, similar results were observed when MKN45 cells were cultured in the CM supplemented with the mammary epithelial growth supplements (MEGS) for the growth of human epithelial cells (Suppl. Figure 3).

### SRF in fibroblasts promotes the pulmonary metastases of GC cells

**Figure 3:**
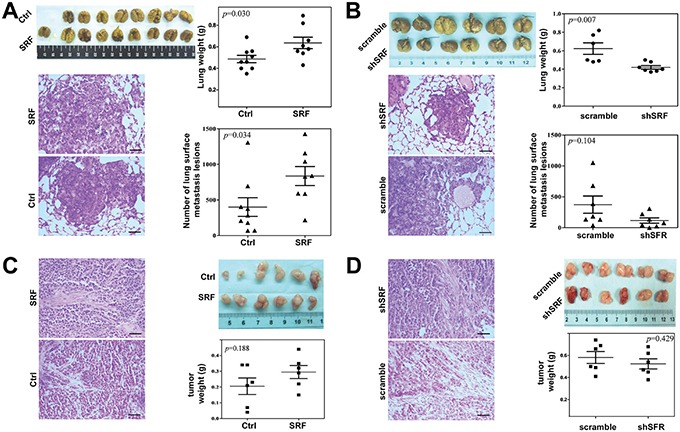
SRF in fibroblasts promotes the pulmonary metastases of gastric cancer cells in NOD-SCID mice **A** and **B.** The average lung weight and number of metastatic nodules on the lung surface were recorded 29 and 21 days after the co-injection of MKN45 cells, and either *SRF*-overexpressing CCD18Co cells or CCD18Co cells treated with shSRF, respectively. **C** and **D.** The tumor xenografts were shown after 16 and 15 days following the co-injection of MKN45 cells and either *SRF-* overexpressing CCD18Co cells or CCD18Co cells treated with shSRF, respectively. The hematoxylin and eosin (H&E)-stained tumor tissues were also shown. Black bar: 200 μm. The data represent the mean ± SD.

To determine whether *SRF* promotes the invasion/metastasis of cancer cells *in vivo*, a SCID mouse model of pulmonary metastases was used. The results showed that the average lung weight and number of metastatic nodules in the lung surface were significantly increased when MKN45 and SRF-overexpressing CCD18Co cells were co-injected into the mouse tail vein (Figure 3A). In contrast, the number of metastatic nodules in the lung surface was significantly decreased when MKN45 and shSRF-knockdown CCD18Co cells were co-injected (Figure 3B).

To investigate the effect of changes of *SRF* expression in fibroblasts on tumor growth *in vivo*, GC xenografts were created by the subcutaneously co-implantation of MKN45 and CCD18Co cells transfected with different vectors. It was found that the growth of the xenografts co-injected with the *SRF*-overexpressing CCD18Co cells was slightly, but not significantly, faster than that of the xenografts containing CCD18Co cells transfected with the pTRIPZ empty vector (Figure 3C). In contrast, the growth of xenografts co-injected with shSRF-transfected CCD18Co cells was slightly, but not significantly, slower than that of xenografts transfected with the scramble control shRNA (Figure 3D). Similar results were obtained when BGC823 cells were subcutaneously co-implanted with SRF-overexpressing NIH3T3 cells (Suppl. Figure 4). In addition, the proliferation of MKN45 (data no shown) and BGC823 (Suppl. Figure 5) cells was not affected by the SRF-overexpressing CM. The upregluation or downregulation of *SRF* expression did not influence the growth or mobility of the CCD18Co and NIH3T3 cells themselves (Suppl. Figure 6). The finding that changes in *SRF* expression in fibroblasts did not significantly affect the proliferation/growth of cancer cells *in vitro* and *in vivo* demonstrates that SRF-enhanced metastasis primarily results from the increased migration/invasion of cancer cells, rather than changes in cell proliferation.

### SRF upregulates *αSMA* and stromal derived factor 1 (*SDF1*) expression in fibroblasts

**Figure 4:**
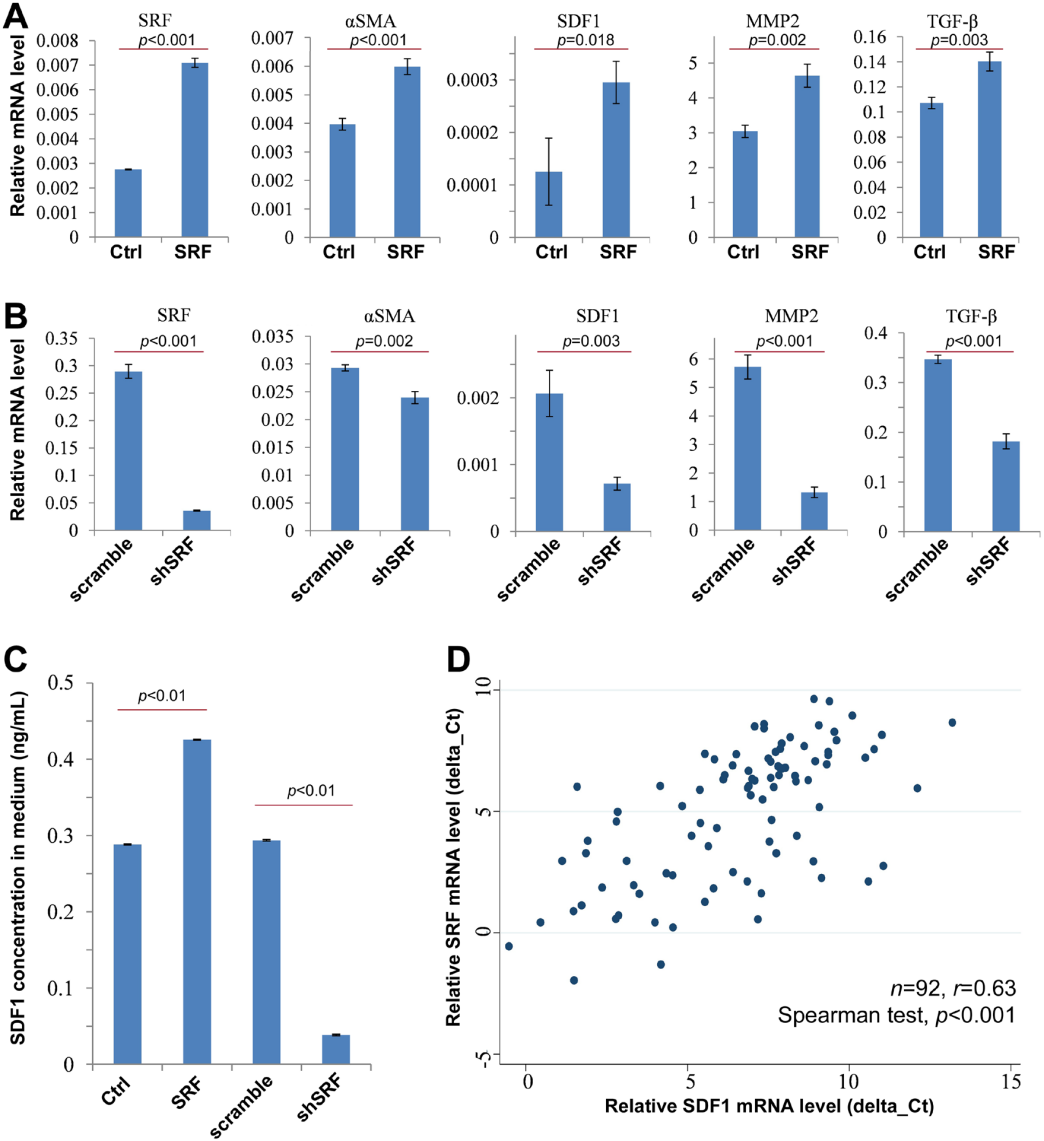
SRF upregulates the expression of αSMA and SDF1 **A** and **B.** The results from qRT-PCR experiments shows the mRNA levels of *SRF*, *αSMA*, *SDF1*, *MMP2*, and *TGF-β* in CCD18Co cells stably infected with the SRF or shSRF pTRIPZ vectors 24 hrs after doxycycline induction; **C.** The concentration of SDF1 in the CCD18Co conditioned medium was determined by ELISA. **D.** qRT-PCR revealed a significant correlation between SRF and SDF1 mRNA levels in GC tissues. The data represent the mean ± SD.

To study the mechanism by which SRF in fibroblasts increases the migration of cancer cells, qRT-PCR was used to detect changes of the expression of a set of metastasis-related genes, including *αSMA*, *SDF1*, matrix metallopeptidase 2 (*MMP2*), and tumor growth factor-β (*TGF-β*). The results showed that the mRNA levels of these genes were significantly increased in CCD18Co cells stably transfected with the *SRF* expression vector and treated with doxycycline when compared with those transfected with the control vector (Figure 4A). In contrast, the expression levels of these genes were significantly decreased in CCD18Co cells stably transfected with the shSRF vector (Figure 4B). Similar results were also observed in NIH3T3 cells (data not shown).

SDF1 is a major chemokine secreted by inflammatory cells and tissue myofibroblasts that play an important role in cell migration and cancer metastasis. Therefore, the possible role of SDF1 in the SRF-enhanced migration of cancer cells was further studied. As shown in Figure 4C, the concentration of SDF1 in CCD18Co conditioned medium was sharply increased 24 hrs after doxycycline treatment. Moreover, *SRF* mRNA levels were positively and significantly correlated with *SDF1* mRNA levels in GC tissues (n=92; *r*=0.63, *p*<0.0001; Figure 4D). Through mining the results of SDF1 IHC analysis using GC tissue microarray in the human protein atlas (www.proteinatlas.org) [27], we found that strong nucleus SDF1-expressing mainly resides in the stromal cells in GCs (Suppl. Figure 7). Together, these results suggest that SRF not only upregulates *SDF1* expression in fibroblasts, but also promotes SDF1 secretion from the fibroblasts into the cancer stroma.

### SRF in fibroblasts enhances cell migration in an SDF1-CXCR4-dependent manner

**Figure 5:**
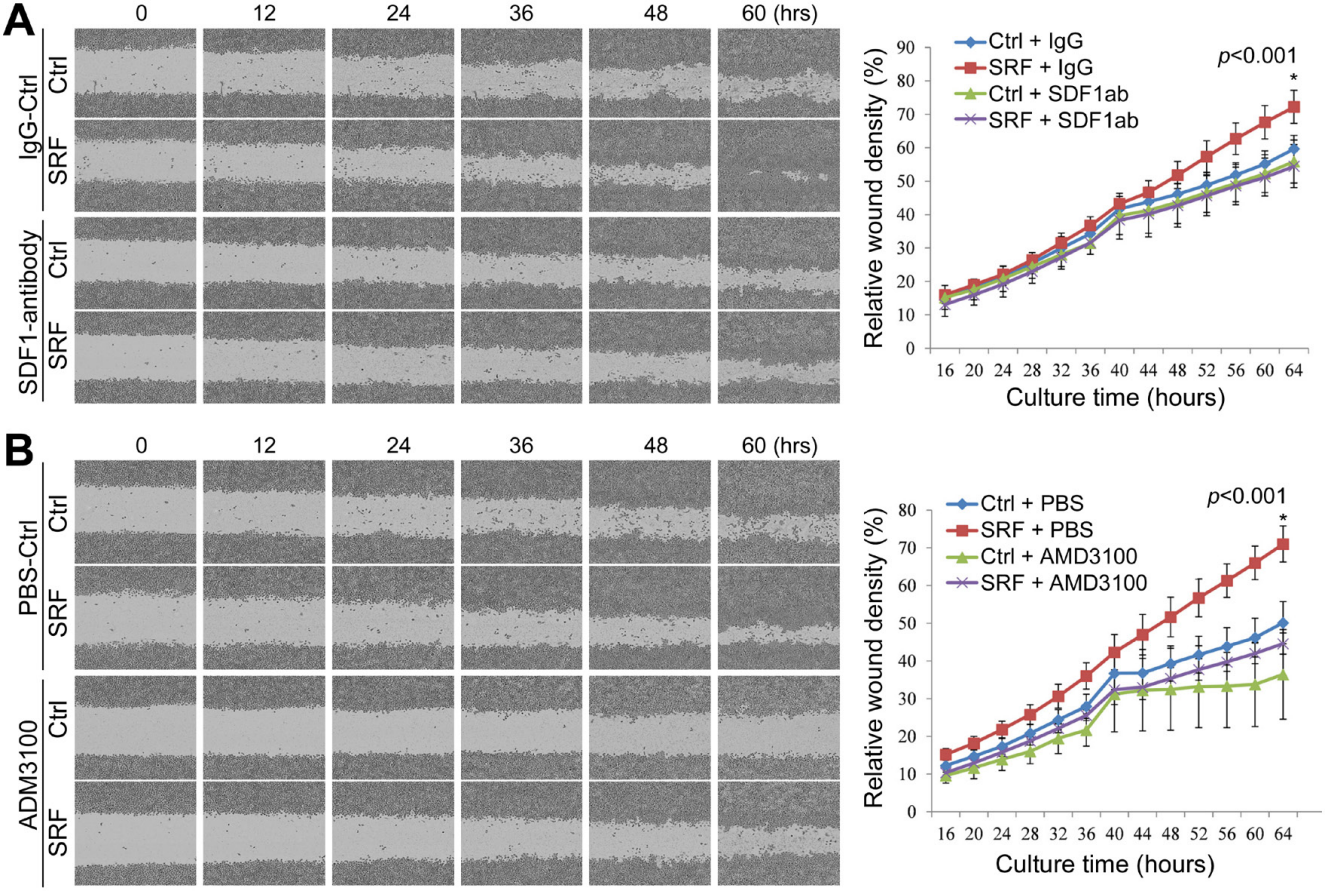
Treatment with anti-SDF1 antibody and AMD3100 reverses the SRF-enhanced migration of MKN45 cells in a wound-healing assay The relative wound density was recorded over time using the IncuCyte ZOOM™ live-cell imaging platform. The migration capacity of MKN45 cells cultured in conditioned medium from CCD18Co cells stably transfected with the SRF or control vector was detected over time. **A.** MKN45 cells were cultured in the conditioned medium with or without an anti-SDF1 antibody (SDF1ab). **B.** MKN45 cells were cultured in conditioned medium with or without AMD3100. Each value in the right charts is the average value of 6 wells. The data represent the mean ± SD.

To investigate the possible role of fibroblast-secreted SDF1 in the SRF-enhanced migration of cancer cells, the conditioned medium from SRF-overexpressing fibroblasts (CCD18Co or NIH3T3) was treated with normal rabbit IgG or an anti-SDF1 neutralizing antibody (final conc. 5.0 μg/mL) and then used to co-culture MKN45 cells. The wound-healing assay showed that treatment with the anti-SDF1 antibody completely reversed the SRF-enhanced migration capacity of MKN45 cells, whereas no change in migration was observed following treatment with the IgG control (Figure 5A).

**Figure 6:**
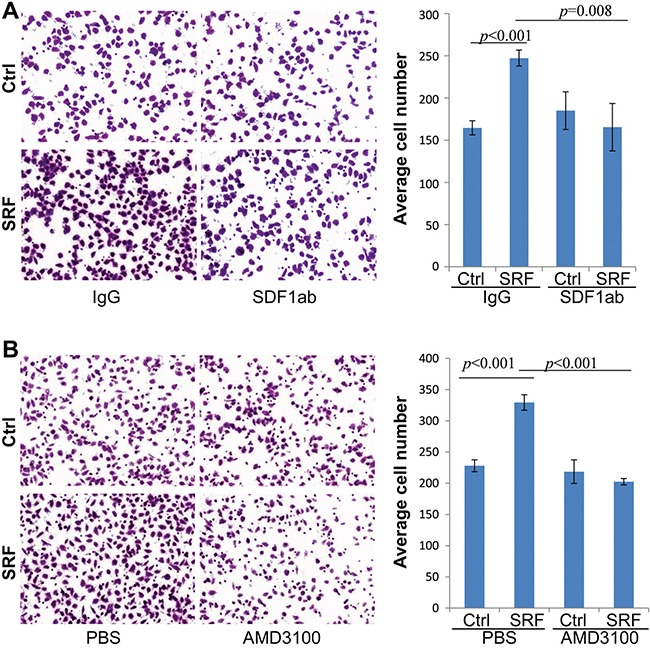
Treatment with anti-SDF1 neutralizing antibody and AMD3100 abolishes the SRF-enhanced migration of MKN45 cells in a typical transwell assay **A** and **B.** The migration capacity of MKN45 cells cultured in the conditioned medium from CCD18Co cells stably transfected with the SRF or control vector and treated with anti-SDF1 antibody (SDF1ab) or AMD3100. The images were captured 12 hrs after co-culture. The data represent the mean ± SD (n=3).

CXCR4 is the SDF1 receptor and actively expressed in MKN45 and BGC823 GC cells, but not in CCD18Co nor in MDA-MB-435S cells, as determined by RT-PCR (Suppl. Figure 8). We added AMD3100, a CXCR4-specific inhibitor, into the CM. Interestingly, the SRF-enhanced migration of MKN45 cells was also completely abolished by the addition of AMD3100 (final conc. 1.0 μg/mL) to the co-culture medium as determined by live cell imaging analysis (Figure 5B). Furthermore, the migration of MDA-MB-435S cancer cells, in which *CXCR4* gene is not expressed, was not affected by the CM from SRF-overexpressing fibroblasts (Suppl. Figure 9). In addition, the proliferation of MKN45 cells was not affected by the anti-SDF1 antibody or AMD3100 treatment (Data not shown). Similar results were observed in Transwell assays using both MKN45 and BGC823 cells (Figure 6 and Suppl. Figure 10). These results indicate that SRF promotes cell migration in an SDF1-CXCR4 dependent manner.

## DISCUSSION

As a master transcription factor that regulates the expression of over 200 genes, SRF is thought to promote cancer metastasis through a number of pathways [4–18]. However, the exact mechanism by which SRF promotes cancer metastasis *in vivo* is not well characterized. SDF1-CXCR4 is also one of important signaling pathways involved in cancer progression [21–23]. In the present study, we demonstrated for the first time that SRF is overexpressed in stromal cells in GC tissues, and promotes migration/invasion of GC cells by facilitating myofibroblast-cancer cell crosstalk in an SDF1-CXCR4 dependent manner.

The role of stromal cells in cancer development has long been underestimated. Fortunately, this situation is changing. It has been reported that the upregulation of *SRF* expression in GC cells promotes GC invasion/metastasis, and *SRF* is primarily expressed in cancer cells in GC tissues [18]. However, we used both IHC and IHF to characterize *SRF*-expressing cells and found that most cells positively stained with anti-SRF antibodies in GC tissues were stromal fibroblasts and smooth muscle cells. Moreover, quantitative RT-PCR analysis confirmed that the level of *SRF* mRNA in the micro-dissected stromal cells was two times of that in GC cells. In addition, using the public available human protein atlas data (www.proteinatlas.org/ENSG00000112658-SRF/cancer/tissue/stomach+cancer), we also found that the SRF IHC staining (antibody cat.# HPA001819 and CAB005416) was strong in the nucleus of many stromal cells, but was weak in the cytoplasm of some glandular epithelial GC cells in GC tissue microarray [27]. Although we used a different SRF-specific antibody in the immnostaining assays, the one conceivable explanation for this discrepancy is that most research efforts have focused on changes of gene expression in cancer cells rather than on alterations occurring in cancer stromal cells.

SRF not only promotes the differentiation of myofibroblast from other cells including fibroblasts through trans-differentiation [25], but also plays an important role in the formation of granulation tissue during the healing of rat gastric ulcers [28]. In the present study, we found that SRF expression primarily resides in the αSMA-expressing fibroblasts in the lamina propria of the gastric mucosa, indicating these SRF-expressing stromal cells are periglandular myofibroblasts. Furthermore, our data demonstrated that the SRF expression level in the metastatic GCs was significantly higher than that in the non-metastatic GCs. Most importantly, SRF overexpression in fibroblasts significantly enhanced the migration/invasion of GC cells *in vitro* and *in vivo*. Therefore, SRF contributes to GC metastasis through its role in the crosstalk between cancer cell and fibroblasts, which is consistent with our recent finding that the risk of GCs metastasis is often decreased when SRF expression is silenced by DNA methylation in SM tissues, but not in GC tissues [26].

The roles of the SDF1-CXCR4 axis in cancer metastasis have been extensively studied [29, 30]. The SDF1-CXCR4 axis is known to be important in angiogenesis and to promote local and distal metastasis and cell survival [23, 29, 31]. SDF1 secreted by stromal myofibroblasts stimulates tumor growth and angiogenesis in invasive human breast carcinomas [23]. However, the relationship between SRF and SDF1-CXCR4 has not previously been reported. In our study, we found that *SDF1* (and other metastasis-related genes) expression level in the fibroblast cell lines CCD18Co and NIH3T3 was induced by *SRF* overexpression and decreased by shRNA knockdown of SRF. Moreover, the addition of a SDF1-specific antibody or a specific inhibitor of the SDF1 receptor CXCR4 completely reversed the SRF-enhanced migration of GC cells. Knockdown of CXCR4 depleted the effect of CCD18Co-SRF conditioned medium on the migration of the MDA-MB-435S control cells. These results suggest that the SDF1-CXCR4 axis plays crucial roles in SRF-mediated cancer cell-fibroblast crosstalk. Further evaluation whether other metastasis-related genes is involved in this crosstalk is warranted.

In conclusion, SRF in fibroblasts promotes the migration/metastasis of GC cells. The SDF1-CXCR4 axis may play a crucial role in the SRF-mediated crosstalk between cancer cells and fibroblasts, and be a promising therapeutic target.

## MATERIALS AND METHODS

### Animals

Four to six week-old female NOD-SCID mice were purchased from HFK Bio-technology (Beijing, China) and observed for one week before experiments were performed. All of the mice were specific pathogen free (SPF) and were maintained in a germ-free environment under the approval of the Administration Committee on Experiment Animals, Peking University Cancer Hospital and Institute.

### Human gastric specimens

Frozen GC and the corresponding surgical margin tissue specimens were obtained from 50 inpatients, including 25 patients with metastatic GC and 25 patients with non-metastatic GC, at Peking University Cancer Hospital. The Institutional Review Boards of the Peking University Cancer Hospital and Institute approved the study, and all of the patients gave written informed consent.

### *SRF* expression plasmids and cell lines

The human full-length SRF coding sequence (containing exon-5) was amplified using the PCR primer set forward: 5′-tcgag cgcca tgtta ccgac ccaag-3′ and reverse: 5′-gcggg cggat cattc actct tgg-3′, and cDNA was synthesized using total RNA extracted from the human GC cell line MGC803. The mouse full-length SRF coding sequence was amplified using the PCR primer set forward: 5′-tgccc cgatt cctcg ctgac ttg-3′ and reverse: 5′-gtgac gggcg gatca ttcac tc-3′, and cDNA was synthesized using total RNA from mouse stomach tissue. The PCR reactions were carried out using the TransTaq® HiFi DNA Polymerase and reaction buffer (TransGen Biotech, Beijing, China). The human and mouse SRF coding sequences were cloned into the *NotI/NHeI* and *MIu1/Age1* restriction sites, respectively, in the inducible lentiviral vector pTRIPZ.

The human GC cell lines BGC823 and MGC803 were kindly provided by Dr. Yang Ke, and the MKN45 cell line was provided by Dr. Youyong Lv. The HEK293FT cell line was provided by Dr. Zhiqian Zhang. The mouse NIH3T3, human breast cancer cell MDA-MB-435S, and fibroblast CCD18Co cell lines were purchased from ATCC. The BGC823, MGC803, and MKN45 cells were cultured in RPMI 1640 medium (Gibco, Grand Island, NY, USA) containing 10% FBS (Gibco) and 1% penicillin-streptomycin (Gibco); the HEK293FT cells were cultured in the DMEM medium with 10% FBS. NIH3T3 cells were cultured in DMEM medium with 10% newborn calf serum (NCS) (PAA Laboratories, Germany); and the CCD18Co cells were cultured in MEM (Gibco) with 15% FBS. The cells that were used in the transfection studies were seeded in medium without antibiotics. All of the cells were maintained at 37°C in a 5% CO_2_ atmosphere. All of the cell lines were tested and authenticated using the Goldeneye20A STR Identifiler PCR Amplification Kit (Beijing Jianlian Genes Technology Co. Ltd) before being used in this study [26].

### Lentiviral infection

Twenty-four hrs before transfection, HEK293FT cells were seeded in 6 cm plates. When they reached a confluence of approximately 40%, the HEK293FT cells were transfected with the pTRIPZ or pTRIPZ-SRF lentiviral vector, the pCMV-VSV-G plasmid, and the pCMV-dR8.91 plasmid (ratio of these plasmids, 4:2:3) using the X-tremeGENE HP DNA Transfection Reagent according to the manufacturer's manual. The cell supernatant was collected, filtered with a 0.22 μm filter, and used to infect CCD18Co or NIH3T3 cells 48 hrs after transfection.

The LV3-NC or LV-shSRF lentiviral vector was used in the same way for *SRF* knockdown experiments. Briefly, HEK293FT cells were transfected with the LV3 puro vector, which contained a short hairpin RNA specifically targeting human *SRF* (shSRF). The sequence of the shSRF was 5′-cgatg tttgcc atgag tat-3′. The LV3 empty vector was used as a negative control. To obtain stably-transfected cells, the infected cells were selected three days after transfection by culturing them in medium containing 0.5 μg/mL puromycin (Sigma, St Louis, MO, USA) for at least two weeks.

### Immunohistochemistry (IHC) and immunohistofluorescence (IHF)

A rabbit anti-human SRF polyclonal antibody (Ab53147, Abcam, Cambridge, MA), a mouse anti-human αSMA monoclonal antibody (Ab7817, Abcam), and a mouse anti-CD34 primary antibody (Invitrogen-Beijing Zhongshan Golden Bridge Biotech) were used as the primary antibodies. Histostain™-Plus Kits (SP-9001, SP-9002 [biotin-labeled goat anti-rabbit or mouse IgG] and SP-9000 HRP-labeled strepto-avidin, ZYMED) and a DAB Kit (ZLI-9018, Invitrogen-Beijing Zhongshan Golden Bridge Biotech) were used to visualize the primary antibody-binding cells according to the manufacture's instructions,. Briefly, paraffin section (4 μm) were dewaxed and rehydrated in xylene and ethanol. The sections were autoclaved in a 10 mM sodium citrate buffer containing 0.05% Tween 20 (pH6.0) for 3 min for antigen retrieval and then immersed in 3% H_2_O_2_ for 10 min to block endogenous peroxidase. After being submerged in goat blocking serum for 30 min, the sections were incubated with the anti-SRF (1:50) or anti-αSMA antibody (1:250) overnight at 4°C. The PBS-washed sections were further treated with the Histostain™-Plus and DAB Kits and counterstained with hematoxylin. Normal rabbit IgG was used as the negative control. For IHF, the anti-SRF or anti-αSMA primary antibody-treated slides were washed with PBS, incubated with TRITC-labeled anti-rabbit IgG (1:100; Ab6725, Abcam) or FITC-labeled anti-mouse IgG (1:100; ZF-0312, Invitrogen-Beijing Zhongshan Golden Bridge Biotech) at 22°C for 30 min, and then counterstained with DAPI (1μg/mL; Sigma). The fluorescent images (red: SRF, green: αSMA, and blue: DNA) were captured using a confocal microscope.

### Laser microdissection

The fresh gastric cancer tissue was embedding with optimum cutting temperature (OTC) and cutted into 10 μm. section, then the frozen sections was stained with hematoxylin and eosin, and the stromal cells and the gastric cancer cells were respectively separately captured from the tissues using the Laser Micro Dissection System (Leica LMD 7000).

### Western blotting

The cells were collected and lysed to obtain protein lysate. The resulting proteins were then electrophoresed through a 10% SDS-PAGE gel and transferred onto a PVDF membrane. After blocking with 5% non-fat milk overnight at 4°C, the membrane was incubated with the primary antibody (anti-SRF, Abcam, ab53147; anti-αSMA, Abcam, ab7817; anti-SDF1, clone K15C, MABC184, Millipore, Merck KGaA, Darmstadt, Germany; or anti-GAPDH, Protein Tech, 60004-1, China) for 1 hr at room temperature. The membrane was then washed 3 times with PBST (PBS with 0.1% Tween-20). After washing, the membrane was incubated with the corresponding horseradish peroxidase-conjugated goat anti-mouse or rabbit IgG at room temperature for 1 hr. The signals were visualized using the Immobilon Western Chemiluminescent HRP Substrate Kit (Millipore, USA).

### CXCR4 inhibition

To block the SDF1 signaling pathway, NMK45 cells were treated with AMD3100 (final concentration, 1.0 μg/mL) (Sigma, St Louis, MO, USA).

### Preparation of conditioned medium

CCD18Co or NIH3T3 cells that had been stably transfected with the SRF vector were cultured in the appropriate DMEM medium without serum or supplemented with the mammary epithelial growth supplements (MEGS) (Gibco) containing 1.0 μg/mL doxycycline for 24 hrs, and the medium was subsequently collected and centrifuged for 5 min at 3000 rpm. The resulting supernatant was either used immediately or frozen at −80°C until needed.

### ELISA

Serum-free cell culture supernatants were collected from the fibroblasts after incubation for 24 hrs, and the human or mouse SDF1-alpha Quantikine ELISA (R&D Systems) was performed according to the manufacturer's instructions. These experiments were performed in triplicate.

### Transwell migration, Matrigel invasion, and co-culture

A cell suspension (2×10^5^ cells/mL) in 200 μl serum-free medium was seeded in the well inserts of transwell plates with a pore size of 8 μm (Corning, Lowell, MA, USA). To study the effect of SRF inhibition on the migration of cancer cells, 1×10^5^ cells in 200 μl serum-free medium supplemented with 1.0 μg/mL AMD3100 or vehicle (PBS) were seeded in the well inserts. Serum-containing medium (10% FBS) or conditioned medium was placed in the well below. After incubation for 24 hrs at 37°C and 5%CO_2_, the cells in the upper chamber were removed, and the cells that had moved to the lower chamber were fixed with 2% formalin and stained with crystal violet. The stained cells were then photographed and counted. For the invasion test, the transwell insert was coated with 80 μl Matrigel (diluted with RPMI 1640 Medium at ratio of 1:6) (BD Bioscience, San Jose, CA). Once the Matigel solidified, suspension of 4×10^4^ cells was plated, and the staining was carried out at 48 hrs later. Triplicate transwell inserts were used for each treatment.

### Live content kinetic imaging platform analysis

The live content kinetic imaging platform (IncuCyte Zoom, Essen BioSci, USA) was used to dynamically detect the proliferation and migration of MKN45 and BGC823 cells. The phase object confluence (%) was used to generate a cell proliferation curve. The relative wound density, a measure (%) of the density of the wound region relative to the density of the cell region, was used as the metric for cell migration in the wound-healing assays as stated in the user manual.

### Cancer cell co-injections

For co-injection, 1×10^6^ MKN45 or BGC823 cells and 5×10^5^ transfected CCD18Co or NIH3T3 cells were mixed and subcutaneously implanted into one leg of each mouse to create tumor xenografts. The corresponding control cells were simultaneously injected into the opposite side of the same mouse to serve as the negative control. The mice were given distilled, sterile water containing 0.4 mg/mL doxycycline. When the tumor volume reached 1 cm^3^, all of the animals were scarified, and the tumors were excised and weighed.

To produce pulmonary metastasis tumor nodules, the doxycycline-treated mice were i.v. injected through the lateral tail vein with 1×10^6^ cancer cells mixed with 5×10^5^ fibroblasts in 0.15 mL PBS. The body weights of the mice were monitored over time. When the weights began to sharply decline, the mice were killed. The lungs from the mice were weighed and fixed in Bouin's solution, and the number of peripheral tumor nodules was determined.

### Quantitative RT-PCR (qRT-PCR) and nested qRT-PCR

Total RNA was extracted using the Ultrapure RNA Kit (Beijing ComWin Biotech Co., Ltd, China) and reverse-transcribed using the first-cDNA synthesis kit (Transgen Co, Beijing, China) according to the user manual. The qRT-PCR reactions were performed using the Power SYBR Green PCR Master Mix (Fermentas, Glen Burnie, MD) on the ABI-7500 or ABI-7500 Fast platform. *GAPDH* was used as an internal control. The primers used are shown in Suppl. Table 1.

The level of *SRF* mRNA in the micro-dissected cells was analyzed using nested qRT-PCR assay due to the low efficiency of regular qRT-PCR amplification using the damaged templates extracted from the captured cells. The regular RT-PCR products (166bp) for SRF mRNA were used as the template in the nested qRT-PCR. The nqRT-hSRF primers were used (Suppl. Table 1). *Alu* transcripts were used as reference [32].

### Statistical analysis

Statistical analysis was performed with the Student's t-test. The Spearman test was used to analyze the correlation between the different groups. Two-sided *P*-values of 0.05 or less were considered to be statistically significant.

## SUPPLEMENTARY FIGURES AND TABLE


