# Supplementary material for: SRF promotes gastric cancer metastasis through stromal fibroblasts in an SDF1-CXCR4-dependent manner
Source: Oncotarget. 2016 Jun 14;7(29):46088–99. doi: 10.18632/oncotarget.10024 (PMC5216783; doi:10.18632/oncotarget.10024)
Supplement: Supplementary file 1 [file oncotarget-07-46088-s001.pdf]

SRF promotes gastric cancer metastasis through stromal fibroblasts in an SDF1-CXCR4-dependent manner

SUPPLEMENTARY FIGURES AND TABLE

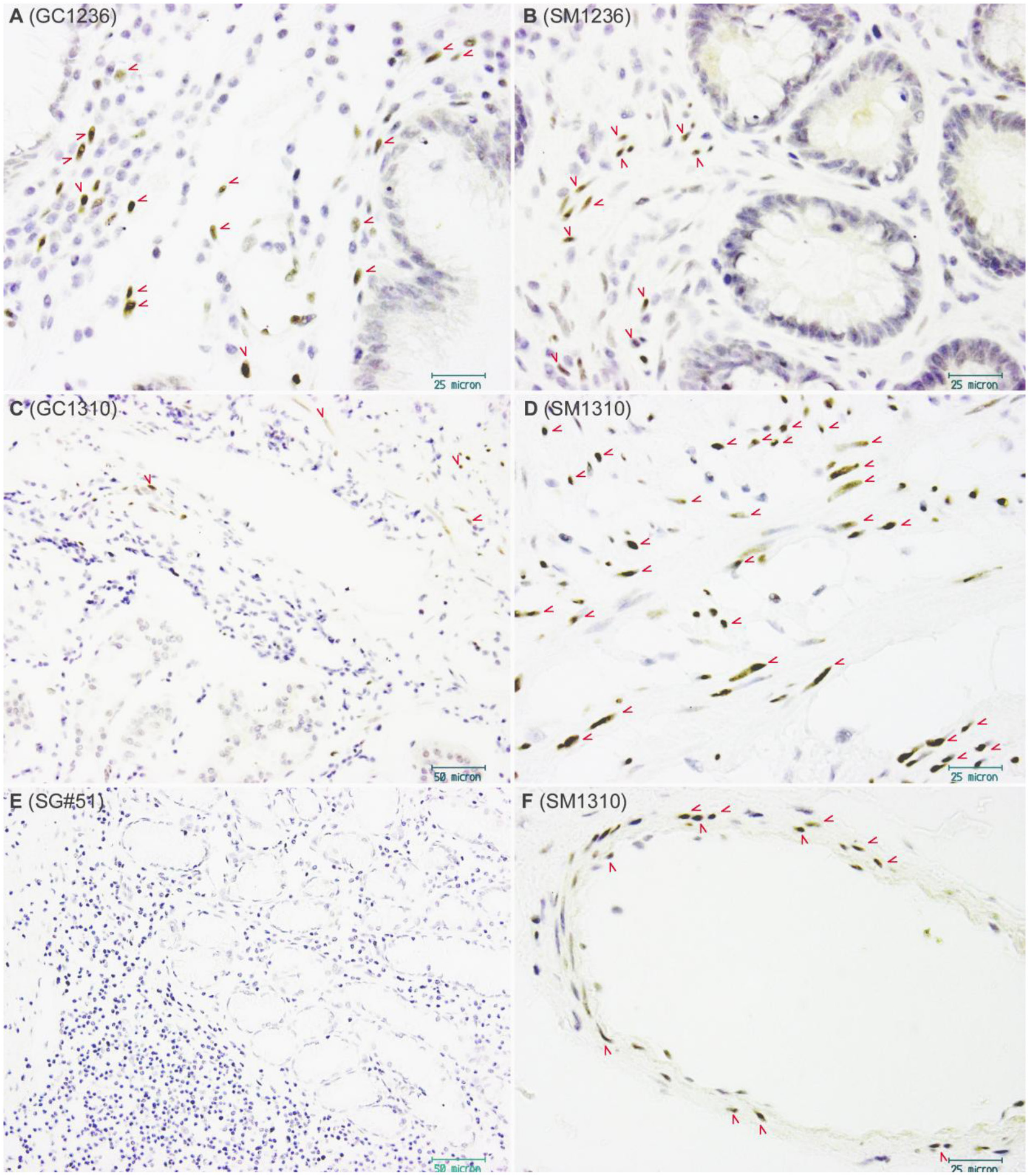

Supplementary Figure S1: IHC Images showing SRF-staining in gastric tissues. (**A/C** and **B/D/F**) Gastric carcinoma (GC) tissues and the corresponding surgical margin (SM) tissues, respectively. (**E**) A superficial gastritis lesion from a non-cancer subject. SRF staining (arrow) was observed in the fibroblasts and the smooth-muscle cells in the connective tissues **A-D.** and blood vessels **F.** Very weak cytoplasmic staining was observed in the epithelial cells **B** and **C.** SRF-stained cells were not observed in the mucosa or submucosa of the gastritis lesion **E.**

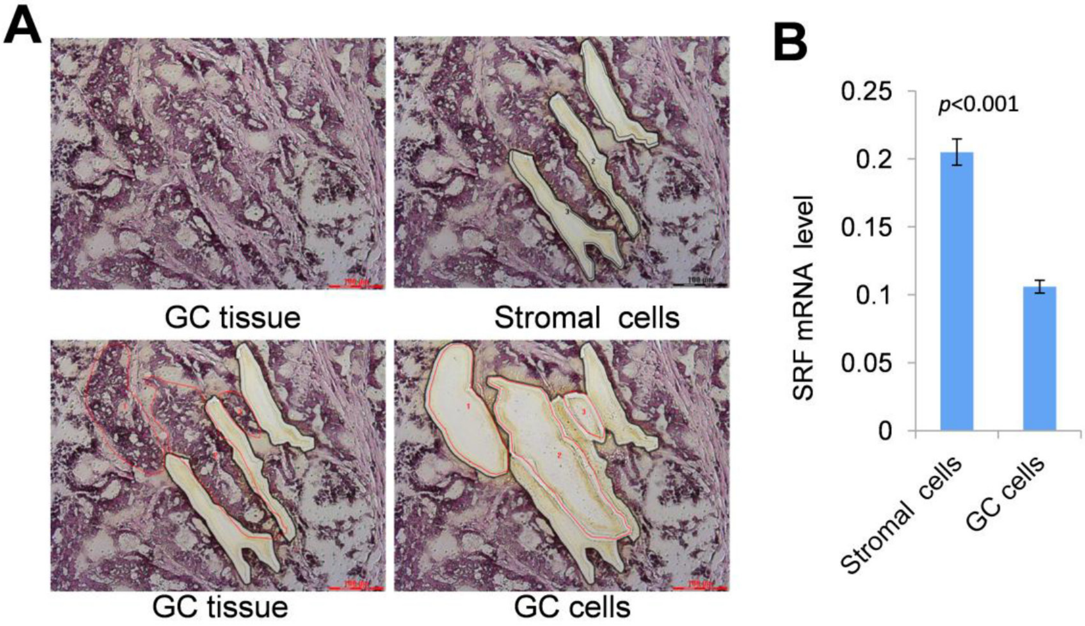

Supplementary Figure S2: Comparison of the levels of *SRF* mRNA in the microdissected GC cells and the stromal cells. A. Images of a representative gastric carcinoma (GC) tissue before and after three stromal and glandular epithelial cell regions were micro-dissected using laser capture microscope system. **B.** Quantitative RT-PCR analysis showed that the level of *SRF* mRNA in the microdissected stromal cells was two times of that in the dissected GC cells.

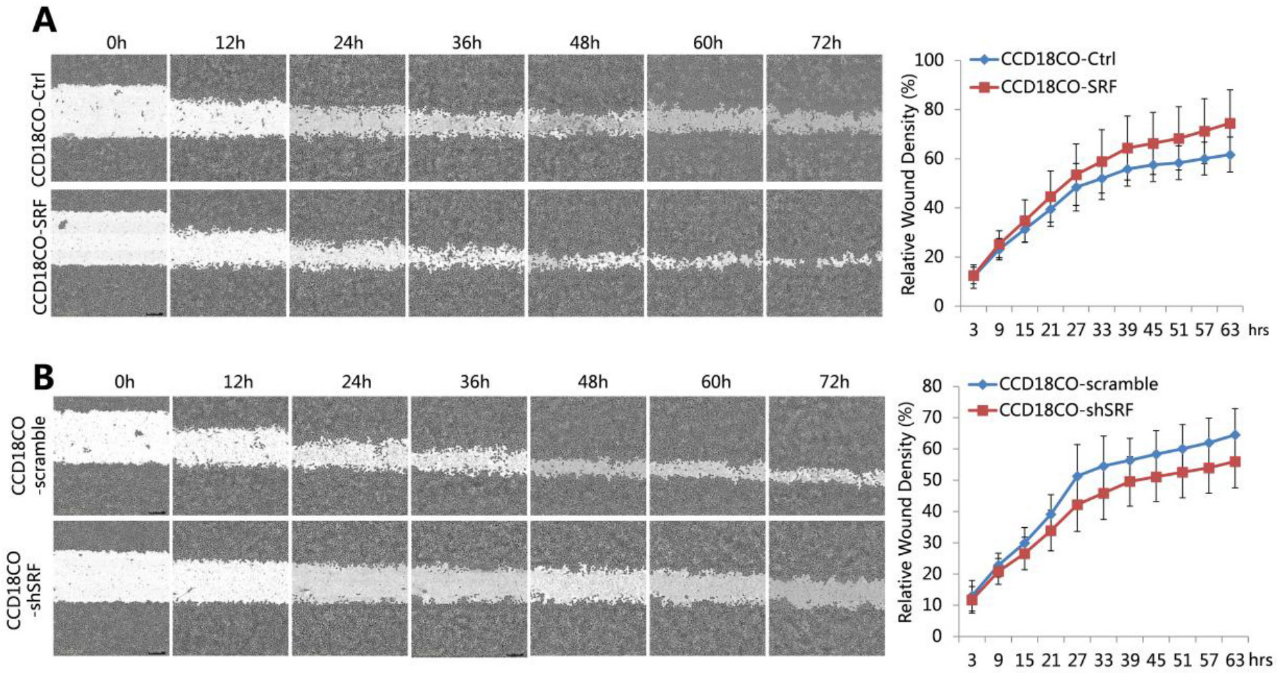

Supplementary Figure S3: SRF in CCD18Co fibroblasts promoted the migration of cultured cancer cells *in vitro*. A and **B.** The migration of MKN45 cells cultured with the conditioned medium with MEGS supplement, as determined using the wound-healing assay. The values from 6 wells were used to calculate the mean and SD at each time point for each treatment.

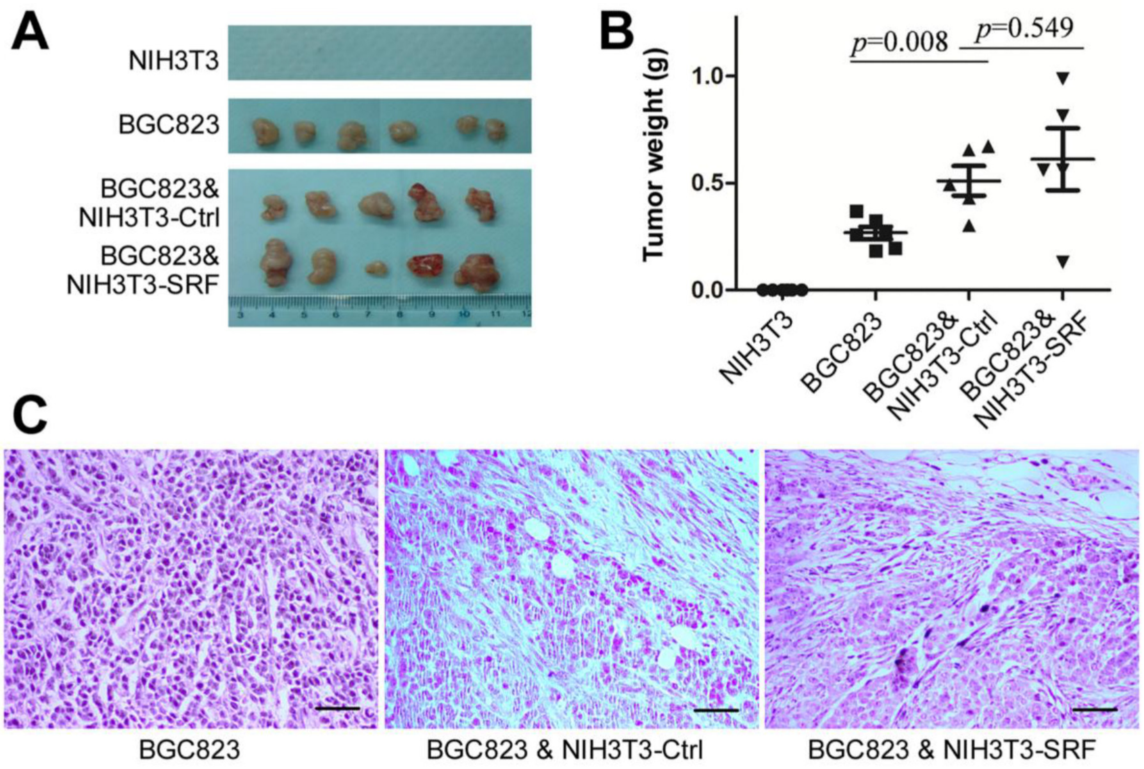

Supplementary Figure S4: The tumor xenografts formed from the co-injection of BGC823 and SRF-overexpressing NIH3T3 cells. The co-injected NIH3T3 cells were stably transfected with the mouse *SRF*-pTRIPZ expression vector. The mice were given distilled, sterile water containing 0.4 mg/mL doxycycline. **A.** The tumor xenografts on experimental day 12. **B.** The average tumor weight; the data represent the mean ± SD. **C.** Hematoxylin and eosin (H&E)-stained tumor tissues; Black bar: 200 µm.

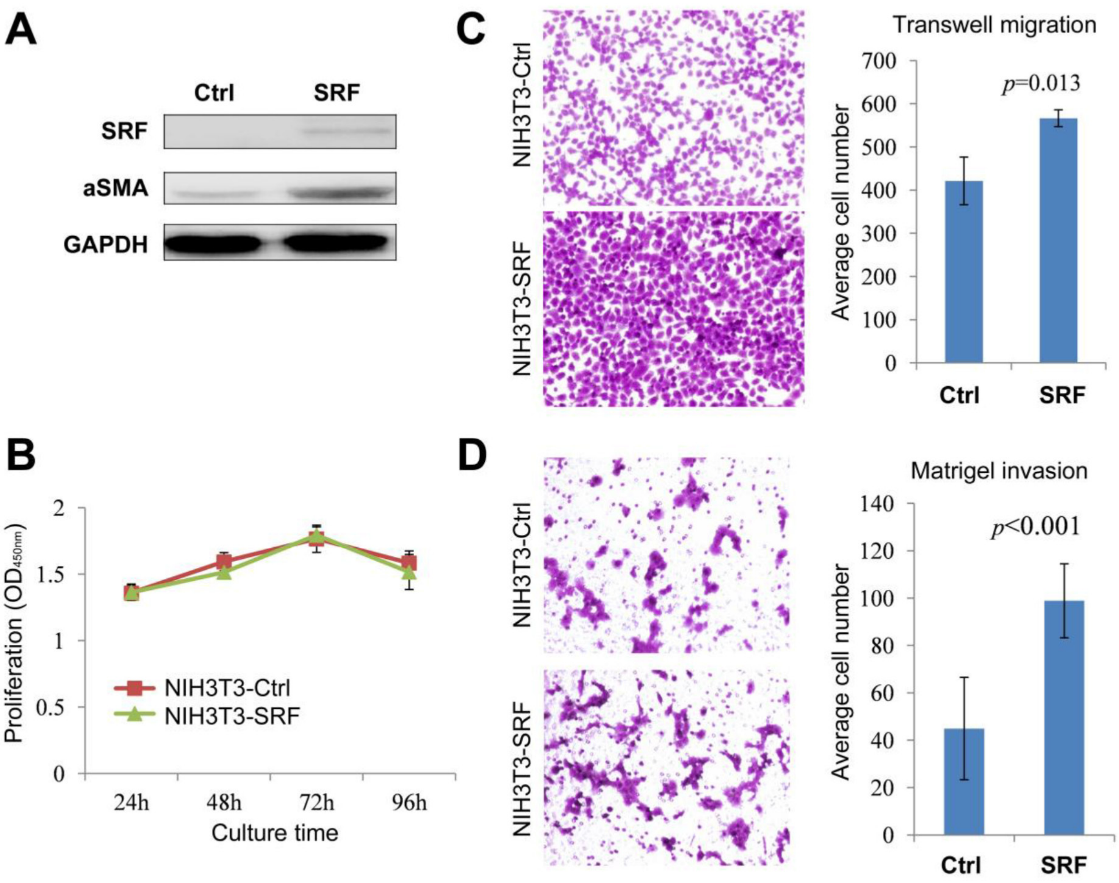

Supplementary Figure S5: Effect of the conditioned medium from *SRF*-overexpressing NIH3T3 cells on the proliferation, migration, and invasion of BGC823 cells. A. Western blot results showing SRF and αSMA expression levels in NIH3T3 cells stably transfected with the mouse *SRF* expression vector. **B.** The proliferation curves of human gastric carcinoma BGC823 cells cultured with conditioned medium from NIH3T3 cells with or without *SRF* overexpression as determined by the CCK8 assay. **C** and **D.** The migration and invasion capacity of BGC823 cells cultured for 18 or 72 hrs with conditioned medium from NIH3T3 cells with or without *SRF* overexpression in the typical transwell migration and Matrigel assays, respectively.

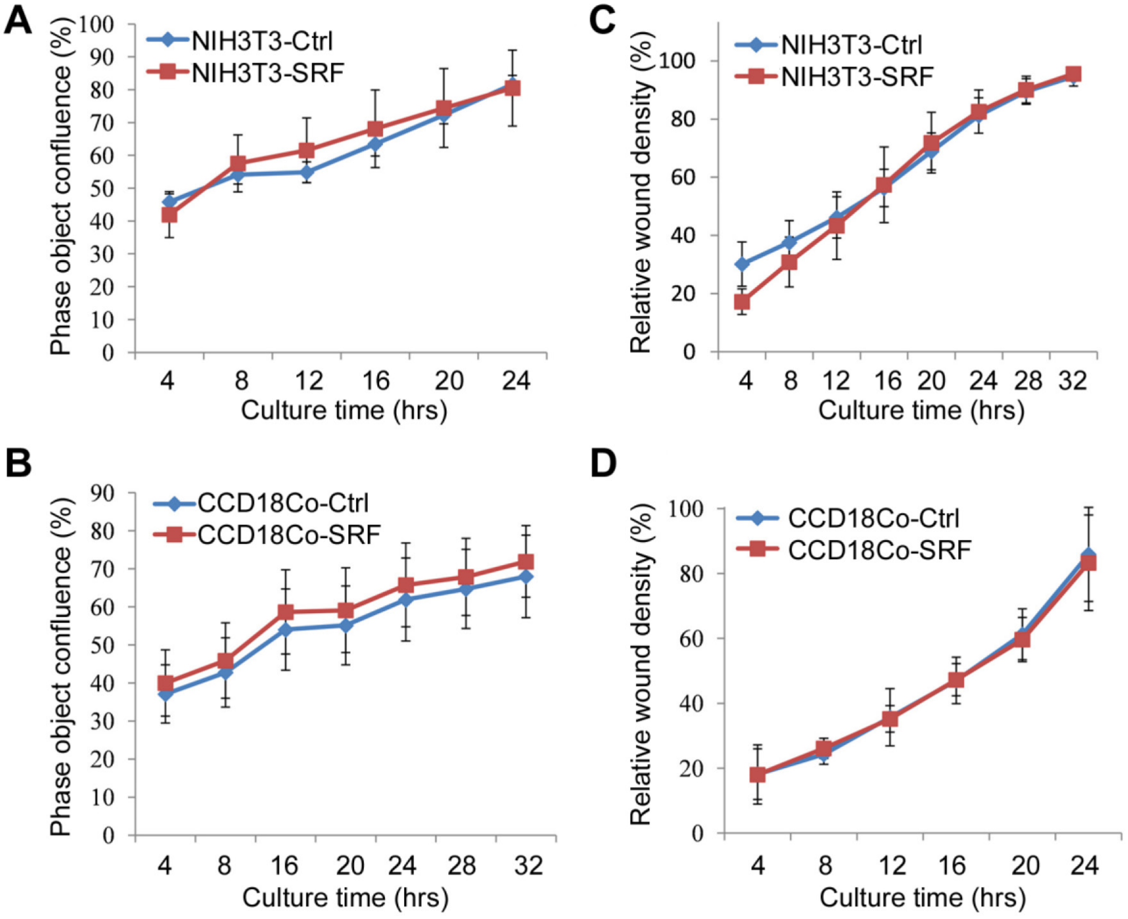

Supplementary Figure S6: Effect of *SRF* overexpression on the proliferation and migration of fibroblasts. A and **B.** The proliferation of NIH3T3 and CCD18Co cells stably transfected with the *SRF* expression or control vector as determined using the live content kinetic imaging system. **C** and **D.** The migration of NIH3T3 and CCD18Co cells stably transfected with the *SRF* expression or control vector.

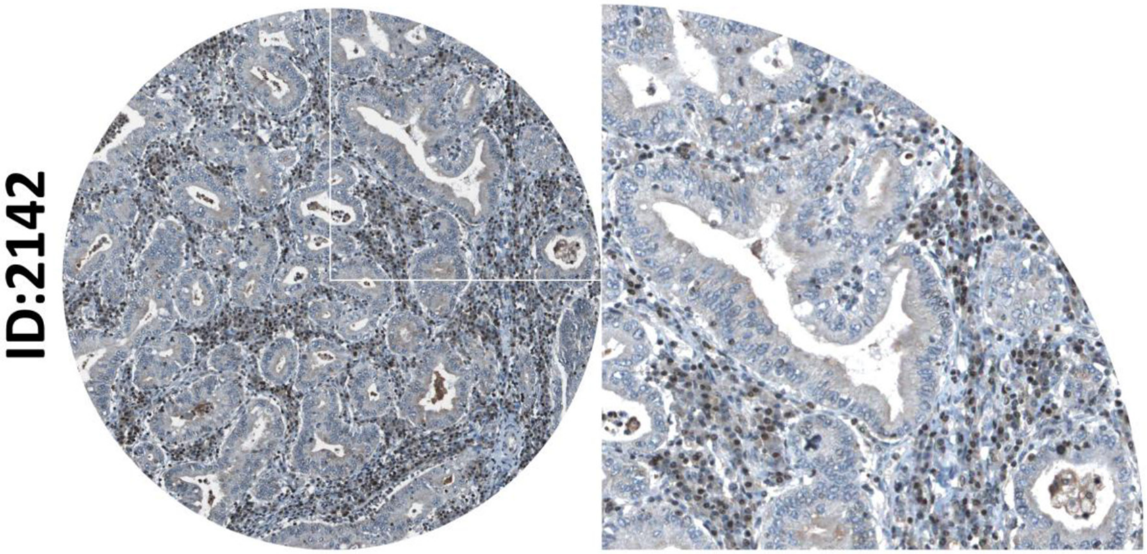

Supplementary Figure S7: Images of SDF1 expressing cells in one representative GC (Patient ID:2142) in IHC analysis using SDF1-specific antibody CAB017564. Strong nucleus staining is observed in many stromal cells. These images are captured from original images downloaded from the web site www.proteinatlas.org [27].

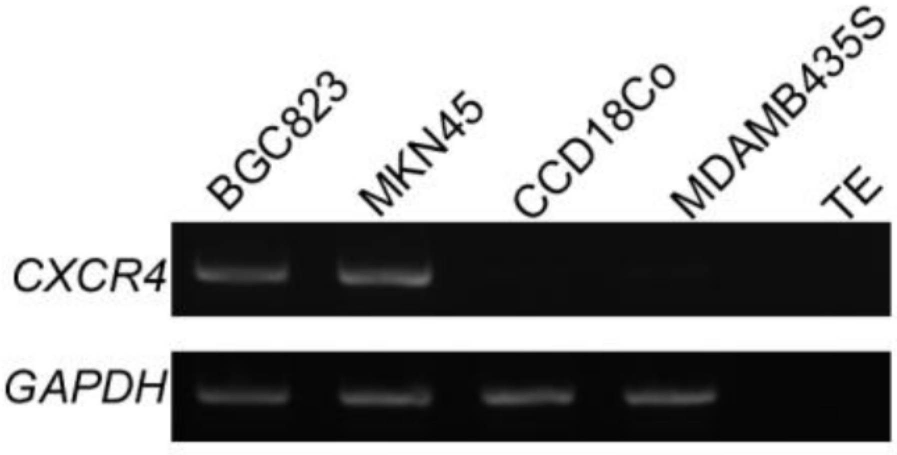

Supplementary Figure S8: RT-PCR analysis showing the expression of the SDF1 receptor CXCR4 in CCD18Co, MKN45, and BGC823 cells. The cDNA sample from MDAMB435S cells is used as the CXCR4 negative control.

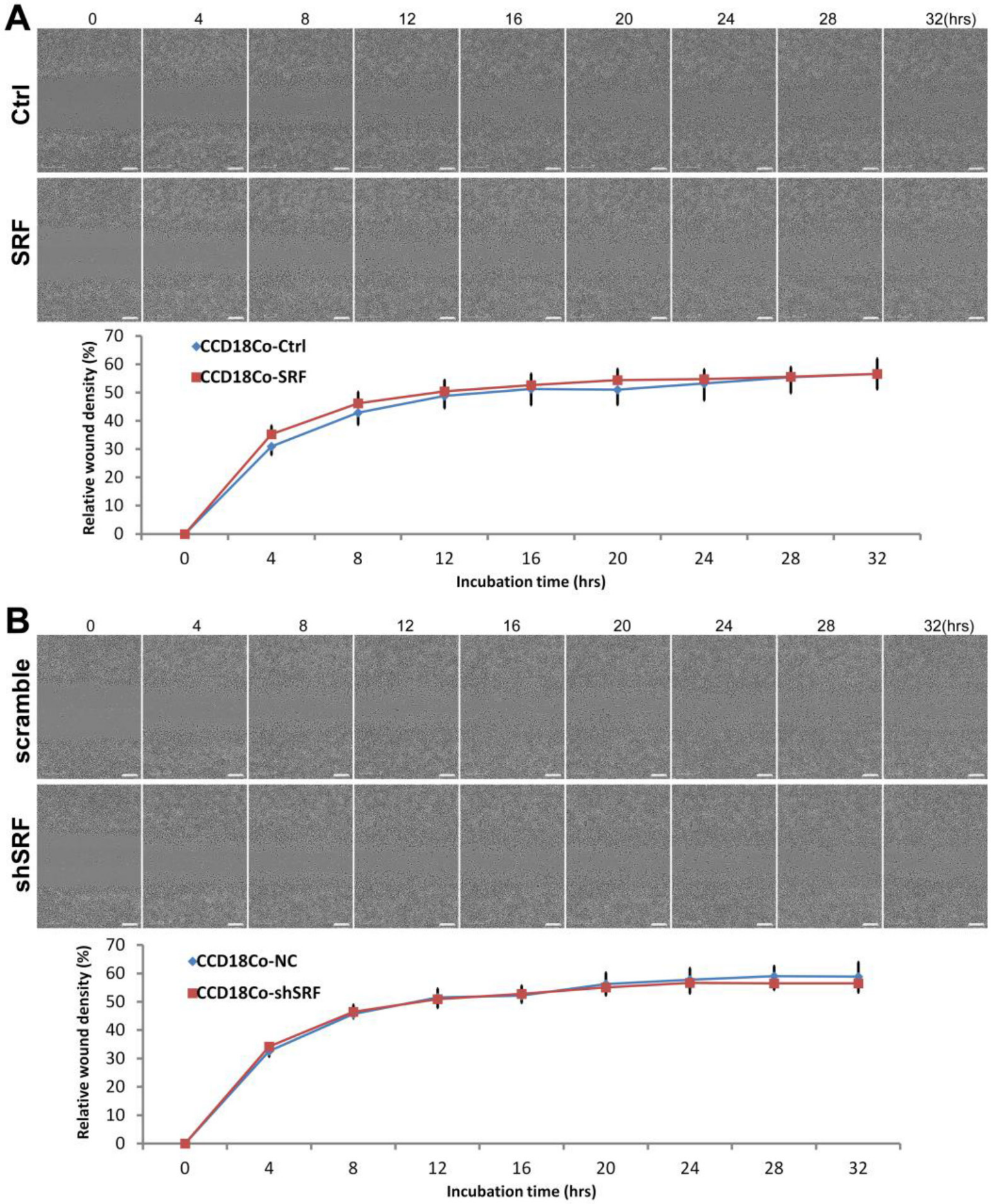

Supplementary Figure S9: SRF in CCD18Co fibroblasts did not promote the migration of cultured MDA-MB-435S cancer cells without CXCR4 expression. The relative wound density was recorded over time using the IncuCyte ZOOM™ live-cell imaging platform. **A.** The migration of MDA-MB-435S cells cultured with different conditioned medium from CCD18Co cells stably transfected with the *SRF* expression or control vector, as determined using the wound-healing assay. **B.** The migration of MDA-MB-435S cells cultured with different conditioned medium from CCD18Co cells stably transfected with the *SRF*-specific shRNA or scramble control vector.

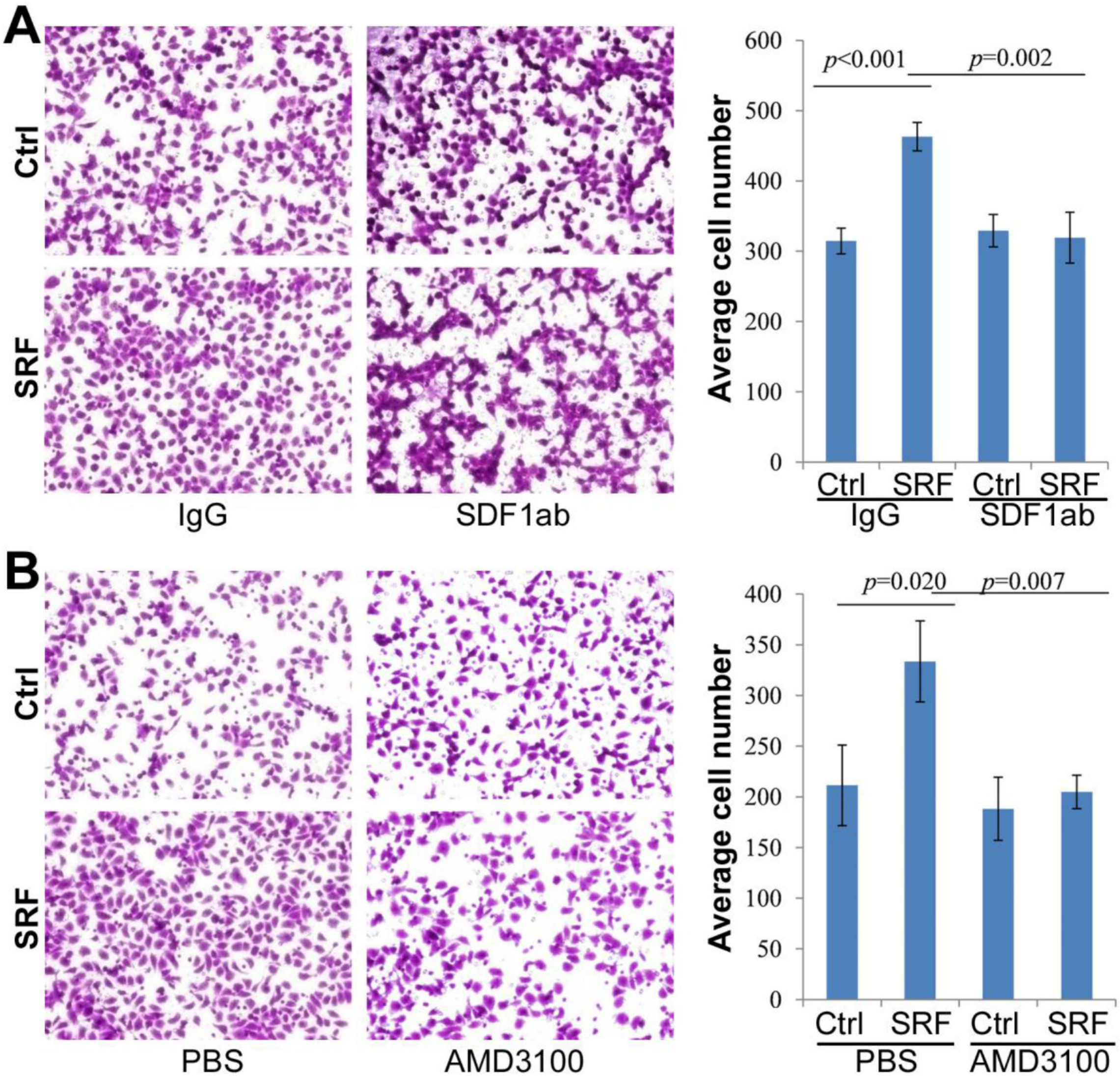

Supplementary Figure S10: Anti-SDF1 neutralizing antibody and AMD3100 reversed the SRF-enhanced migration of BGC823 cells in a transwell assay. A and **B.** The migration capacity of BGC823 cells cultured in conditioned medium from NIH3T3 cells transfected with the SRF or control vector and treated with anti-SDF1 antibody (SDF1ab) or AMD3100. The images are captured after 12 hrs of culture. The data represent the mean ± SD (n=3).

Supplementary Table S1: Oligo sequences of primers used in quantitative RT-PCR analysis

Gene	Primer id	Oligo sequence (5'→3')	Entrez gene	Product size	PCR Tm (°C)
Srf (mouse)	qRT-msrf-F<br>qRT-msrf-R	gcaccgtctgggaccgtgcagatcc<br>gtgctgtgggtggcatccaggttca	20807	133bp	60
SRF (human)	qRT-hSRF-F<br>qRT-hSRF-R	tgctgaatgccttctccca<br>gcctgctgccctatcaca	6722	166bp	56
SRF2 (human)	nqRT-hSRF-F2<br>nqRT-hSRF-R	gtggcgtcccccaggtgtt<br>gcctgctgccctatcaca	6722	100bp	58
α*Sma* (mouse)	qRT-msma-F<br>qRT-msma-R	gtgtgtgacaatggctctgg<br>tggtgatgatgccatgttct	11468	208bp	58
α*SMA* (human)	qRT-hSMA-F<br>qRT-hSMA-R	tgacaatggctctgggctctgtaa<br>ttcgtcacccacgtagctgtcttt	59	141bp	58
c-*Fos* (mouse)	qRT-mcfos-F<br>qRT-mcfos-R	cctgcccccttctcaacgac<br>gctccacgttgctgatgct	14281	71bp	60
c-*FOS* (human)	qRT-hcfos-F<br>qRT-hcfos-R	actaccactcacccgcagac<br>gtgggaatgaagttggcact	2353	104bp	57
Sdf1 (mouse)	qRT-msdf-F<br>qRT-msdf-R	gagagccacatcgccagagc<br>ggatccactttaatttcgggtcaa	20315	133bp	60
SDF1 (human)	qRT-hSDF-F<br>qRT-hSDF-R	gattgtagcccggctgaaga<br>ttcggtcaatgcacacttgt	6387	46bp	60
Tgf-β (mouse)	qRT-mtgfβ-F<br>qRT-mtgfβ-R	ttgcttcagctccacagaga<br>tggttgtagagggcaaggac	21803	183bp	56
TGF-β (human)	qRT-hTGF-β-F<br>qRT-hTGF-β-R	actgcaagtggacatcaacg<br>tgcggaagtcaatgtacagc	7040	218bp	56
CXCR4 (mouse)	qRT-mcxcr4-F<br>qRT-mcxcr4-R	gaccgcctttaccccgatagc<br>acccccaaaaggatgaaggagtc	12767	248bp	60
CXCR4 (human)	qRT-hCXCR4-F<br>qRT-hCXCR4-R	aatcttcctgcccaccatct<br>gacgccaacatagaccacct	7852	367bp	58
Mmp2 (mouse)	qRT-mmmp2-F<br>qRT-mmmp2-R	catgtcgcccctaaaacaga<br>ccatcaaacgggtatccatc	17390	439bp	58
MMP2 (human)	qRT-hMMP2-F<br>qRT-hMMP2-R	cttcttccctcgcaagcc<br>atggattcgagaaaaccg	4313	157bp	60
Gapdh (mouse)	qRT-mgapdh-F<br>qRT-mgapdh-R	tggcaaagtggagattgttgcc<br>aagatggtgatgggcttcccg	14433	156bp	58
GAPDH (human)	qRT-hGAPDH-F<br>qRT-hGAPDH-R	gagatggtgatgggatttc<br>gaaggtgaaggtcggagt	2597	224bp	58
Alu	qRT-Alu-F<br>qRT- Alu -R	gaggctgaggcaggagaatcg<br>tgtcgcccaggctggagtg		88bp	60
